# Incidence of Electric Scooter–Associated Injuries in Finland From 2019 to 2021

**DOI:** 10.1001/jamanetworkopen.2022.7418

**Published:** 2022-04-14

**Authors:** Aleksi Reito, Elina Öljymäki, Mikko Franssila, Ville M. Mattila

**Affiliations:** 1 Center for Musculoskeletal Diseases, Tampere University Hospital, Tampere, Finland; 2 School of Medicine and Health Technology, Tampere University, Tampere, Finland; 3 Emergency Division, Tampere University Hospital, Tampere, Finland

## Abstract

This cross-sectional study examines the incidence of injuries associated with the use of electric scooters in Finland.

## Introduction

Electric scooters (e-scooters) have become an increasingly popular mode of transportation in metropolitan areas around the world, which has led to many e-scooter–related injuries. The most common injuries are head and facial trauma and extremity injuries.^1,2,3,4,5^ Patients with these injuries are predominantly younger men, and substance use is also relatively common among the injured.^3,5^ The characteristics of the injured and the prevalence of the sustained injuries have been reported by numerous authors. We aimed to evaluate the incidence of e-scooter–related injuries.

## Methods

We performed an observational retrospective cross-sectional study of patients admitted to the Tampere University Hospital emergency department (ED) in Finland. The hospital is responsible for providing 24-hour emergency services for a catchment population of 550 000 inhabitants. E-scooters were introduced in April 2019, and until April 2021, 2 companies provided e-scooter rental services within the hospital catchment area. We assessed all patients referred to the ED with e-scooter–related injuries from April 23, 2019, to April 23, 2021. We also contacted both e-scooter companies and requested user data (number of rides, kilometers driven) for the same period. The Tampere University Hospital research director approved this study and waived the requirement for patient informed consent because of its retrospective nature. We followed the STROBE reporting guideline.

A search of the medical records database using e-scooter–related keywords was conducted to identify patients (eAppendix in the Supplement). Injuries were categorized based on their anatomic location and severity. Minor injuries included any laceration, contusion, or superficial injury without an imaging finding. For head injuries, any sign or history of a head trauma without imaging finding was considered as minor. Any imaging finding, such as fracture, dislocation, or hemorrhage, was considered as major. In addition, imaging modalities, treatments, substance use, and discharge status were recorded. The 95% CIs for injury rate were estimated with Poisson distribution using R software, version 2021.09.1.

## Results

We identified 562 patients with 594 ED visits matching the search terms. Of these, 331 patients (335 visits) presented because of an e-scooter-related injury, based on manual abstraction. In total, 147 (44.4%) of the visits occurred between midnight and 6:00 am.

A total of 527 injuries were diagnosed in 331 patients (Table). Any fracture or dislocation occurred in 103 patients (31.1%). Of these, distal radius fracture and clavicle fracture (13 patients [12.6%] each) were the most common.

**Table.** Patient Injury and Treatment Characteristics

Characteristic	Patients, No. (%) (N = 331)
Mean (SD) age, y	29 (9)
Sex	
Men	199 (60.1)
Women	132 (39.9)
Injury	
Head	
Minor	90 (27.2)
Major	13 (3.9)
Face	
Minor	121 (36.6)
Major	17 (5.1)
Upper extremity	
Minor	73 (22.1)
Major	63 (19.0)
Lower extremity	
Minor	79 (23.9)
Major	22 (6.6)
Other	
Minor thoracic	8 (2.4)
Major thoracic	2 (0.6)
Dental	26 (7.9)
Minor abdominal	1 (0.3)
Hearing loss	1 (0.3)
Substance misuse	
No	112 (33.8)
Alcohol	167 (50.4)
Other	4 (1.2)
Delayed presentation to ED	53 (16.0)
Discharge from ED	
Home	316 (95.5)
Hospital ward	17 (5.1)
Intensive care unit	3 (0.9)
Operative treatment	
Distal radius fracture	5 (1.5)
Facial fracture	3 (0.9)
Lisfranc injury	2 (0.6)
Ankle fracture	3 (0.9)
Hand fracture	3 (0.9)
Femoral neck fracture	1 (0.3)
Olecranon fracture	1 (0.3)
Clavicle fracture	1 (0.3)
Wound debridement	2 (0.6)
Patellar tendon rupture	1 (0.3)
Tibial shaft fracture	1 (0.3)

Abbreviation: ED, emergency department.

During the study period, 1 862 778 trips were made and 4 592 549 km were driven on e-scooters. The incidence of any injured riders requiring admittance to the ED during the study period was 18.0 (95% CI, 16.2-20.0) per 100 000 rides and 7.3 (95% CI, 6.6-8.1) per 100 000 km driven. The incidence of patients with major trauma was 5.9 (95% CI, 4.9-7.1) per 100 000 rides and 2.4 (95% CI, 2.0-2.9) per 100 000 km driven.

## Discussion

To our knowledge, no previous study in the peer-reviewed literature has evaluated the incidence of e-scooter–related injuries. A 2020 public authority report estimated that the incidence of these injuries was 20 per 100 000 rides.^6^ User data in the study were gathered from the Austin Transportation Department in Texas without further clarification. The study period was only 3 months with fewer than 1 million rides, whereas our study period was 2 years, and the total number of recorded rides was 2 million, indicating a more robust analysis than those reported previously. The main limitation of this study was its retrospective nature.

Because e-scooters remain popular and the market continues to grow, further studies are needed to evaluate targeted safety measures on e-scooter use. The incidence reported in our study can be used as a reference value for new interventions.

## Notes

Supplement. **eAppendix.** Search Protocol
