# Supplementary material for: Incidence of Electric Scooter–Associated Injuries in Finland From 2019 to 2021
Source: JAMA Netw Open. 2022 Apr 14;5(4):e227418. doi: 10.1001/jamanetworkopen.2022.7418 (PMC9011127; doi:10.1001/jamanetworkopen.2022.7418)
Supplement: Supplement. — eAppendix. Search Protocol [file jamanetwopen-e227418-s001.pdf]

Supplementary Online Content

Reito A, Öljymäki E, Franssila M, Mattila VM. Incidence of electric scooter–associated injuries in Finland from 2019 to 2021. *JAMA Netw Open*. 2022;5(4):e227418. doi:10.1001/jamanetworkopen.2022.7418

eAppendix. Search Protocol

This supplementary material has been provided by the authors to give readers additional information about their work.

© 2022 Reito A et al. *JAMA Network Open.*

eAppendix. Search Protocol

Following search terms were used:

sähköpotku

skuut

e-skoot

potkulau

Terms are in Finnish language. *Sähköpotkulauta* means electric scooter. Sometimes term *e-skootteri* is used as a direct translation from English word e-scooter. *Skuutti* is a vernacular word for e-scooter.

All EHRs from recent years are stored in an operational data store (ODS) format. An SQL query was run with the above search terms in the ODS database. The search was done word by word in all relevant specialty notes. These specialties were emergency medicine, orthopaedics, neurosurgery and intensive care.

Each matched record in the database was manually abstracted to see if it involved an e-scooter related injury.

© 2022 Reito A et al. *JAMA Network Open.*
